# Protocol for a study to evaluate the safety and efficacy of prophylactic negative pressure wound therapy after abdominal perineal resection (VACPAC study)

**DOI:** 10.1371/journal.pone.0331361

**Published:** 2025-08-29

**Authors:** Takuya Takami, Yoshiro Itatani, Yu Yoshida, Nobuaki Hoshino, Ryo Ohno, Ryosuke Okamura, Koya Hida, Kazutaka Obama

**Affiliations:** Department of Surgery, Graduate School of Medicine, Kyoto University, Kyoto, Japan; Jan Biziel University Hospital No 2 in Bydgoszcz: Szpital Uniwersytecki Nr 2 im dr Jana Biziela w Bydgoszczy, POLAND

## Abstract

**Introduction:**

Abdominal perineal resection (APR) is a surgical procedure for rectal cancer that frequently results in perineal wound surgical site infections (SSI), particularly in high-risk patients undergoing preoperative treatment. SSI after APR is associated with prolonged hospital stay, increased medical costs, and delayed initiation of adjuvant chemotherapy, potentially leading to poor prognosis. Although omentoplasty and myocutaneous flap reconstruction have been used to prevent SSI, they are not always feasible. Prophylactic negative pressure wound therapy (pNPWT) has been shown to reduce SSI in high-risk wounds; however, its efficacy in APR remains unclear. This study aims to evaluate the safety and efficacy of pNPWT in preventing perineal wound SSI after APR.

**Methods and materials:**

This is a multi-institutional, single-arm trial investigating pNPWT after APR. The study includes patients undergoing APR for malignant rectal tumors who meet the high-risk criteria for SSI. The intervention involves applying the 3M™ Prevena™ Plus Customizable system immediately after perineal skin closure, maintaining pNPWT for up to 7 days. The primary endpoint is the incidence of perineal wound SSI within 30 days. Secondary endpoints include postoperative complications (Clavien–Dindo Grade III or higher), hospital stay, and completion proportion of pNPWT.

**Discussion:**

This study seeks to provide clinical evidence of the efficacy of pNPWT in reducing perineal wound SSI after APR. Given its ease of application and minimal invasiveness, pNPWT may serve as an effective approach for SSI prevention. These findings may contribute to the establishment of a standardized preventive approach for perineal wound management after APR.

## Introduction

Abdominal perineal resection (APR) is a surgical procedure that removes the rectum and anus, and creates an end colostomy. APR is primarily performed to resect tumors located in the very low rectum and anal canal. Surgical site infection (SSI) after APR often occurs, with a reported incidence rate of 19–27% in all patients and 26–51% in patients who received preoperative treatment [1–4]. Minimally invasive approaches, such as laparoscopy and robot-assisted surgery, have reduced abdominal wound SSI, but perineal wound SSI after APR remains a problem. The accumulation of serous fluid and blood in the large cavity after resection of the pelvic contents and perineum during APR can lead to perineal wound SSI. Additionally, tissue damage and immunosuppression caused by preoperative treatment in patients with advanced cancer also contribute to SSI [5].

SSI leads to prolonged hospital stay, increased costs, and decreased quality of life. Moreover, patients who have undergone APR frequently have advanced cancers requiring adjuvant chemotherapy, and its delay in such patients may lead to a poor prognosis [6]. Thus, prevention of SSI after APR is an important issue.

Until now, omentoplasty and myocutaneous flap reconstruction have been reported as preventive approaches for SSI after APR [7,8]. However, omentplasty can be challenging in cases of inflammation or after omentectomy, and myocutaneous flap reconstruction carries the risk of flap necrosis and prolonged operation time [9]. Therefore, a new approach for preventing SSI after APR is required.

Negative pressure wound therapy (NPWT) is a method that promotes wound healing by applying negative pressure [10], and has been conventionally used for the treatment of refractory open wounds, showing favorable outcomes [11,12]. Recently, prophylactic NPWT (pNPWT) has attracted attention for the prevention of SSI in high-risk patients. By drawing the wound edges together to alleviate tension, reducing subcutaneous hematoma and seroma, and creating a sealed environment that protects against external contamination, pNPWT reduces SSI [13]. There are a few studies examining the efficacy of pNPWT on perineal wounds after APR [14–17], but all of them included small sample sizes. Therefore, the establishment of clear evidence is in demand.

## Materials and methods

### Objectives

This study aimed to evaluate the safety and efficacy of pNPWT after APR in high-risk patients for SSI. We hypothesize that pNPWT reduces the proportion of SSI after APR in high-risk patients.

### Study design

This study is a multi-institutional, single-arm trial. Written informed consent will be obtained from every participant by the treating doctor. The study will be performed in accordance with the Declaration of Helsinki. This multi-institutional trial protocol was approved by the Kyoto University Certified Review Board in January 2025 and the institutional review board of each participating institution before initiating patient recruitment. The principal investigator will oversee patient data management for enrolled institutions through an Electronic Data Capture system. This trial was registered in the Japan Registry of Clinical Trials under jRCT 1052240247, which was publicly posted on 22/01/2025.

### Study population

This study population consists of patients who undergo APR for malignant rectal tumors. The inclusion and exclusion criteria are presented in Table 1.

**Table 1:** Inclusion and exclusion criteria.

Inclusion criteria	Patients undergoing APR for malignant rectal tumors.
	Meet the insurance requirements for the PREVENA incision management system (any of the following). (ⅰ) Undergoing preoperative treatment (Neoadjuvant chemotherapy: NAC, Neoadjuvant chemoradiotherapy: NACRT, Total neoadjuvant therapy: TNT) (ⅱ) BMI ≧ 30 (ⅲ) Hemoglobin A1c ≧ 7.0% in National Glycohemoglobin Standardization Program (ⅳ) Receiving steroid therapy (ⅴ) Chronic maintenance dialysis (ⅵ) Malnutrition
	18 years old and over at the registration, both sexes, an Eastern Cooperative Oncology Group performance status of 0 or 1.
	Written informed consent.
Exclusion criteria	Emergency surgery cases.
	Cases where primary wound closure is undesirable because of contaminated/infected wound.
	Myocutaneous flap reconstruction cases.
	Cases with allergies or hypersensitivity to silver or acrylic adhesives.
	Cases deemed unsuitable for this study by the principal investigator or sub-investigators.

BMI: body mass index.

### Study protocol

**Fig 1:**
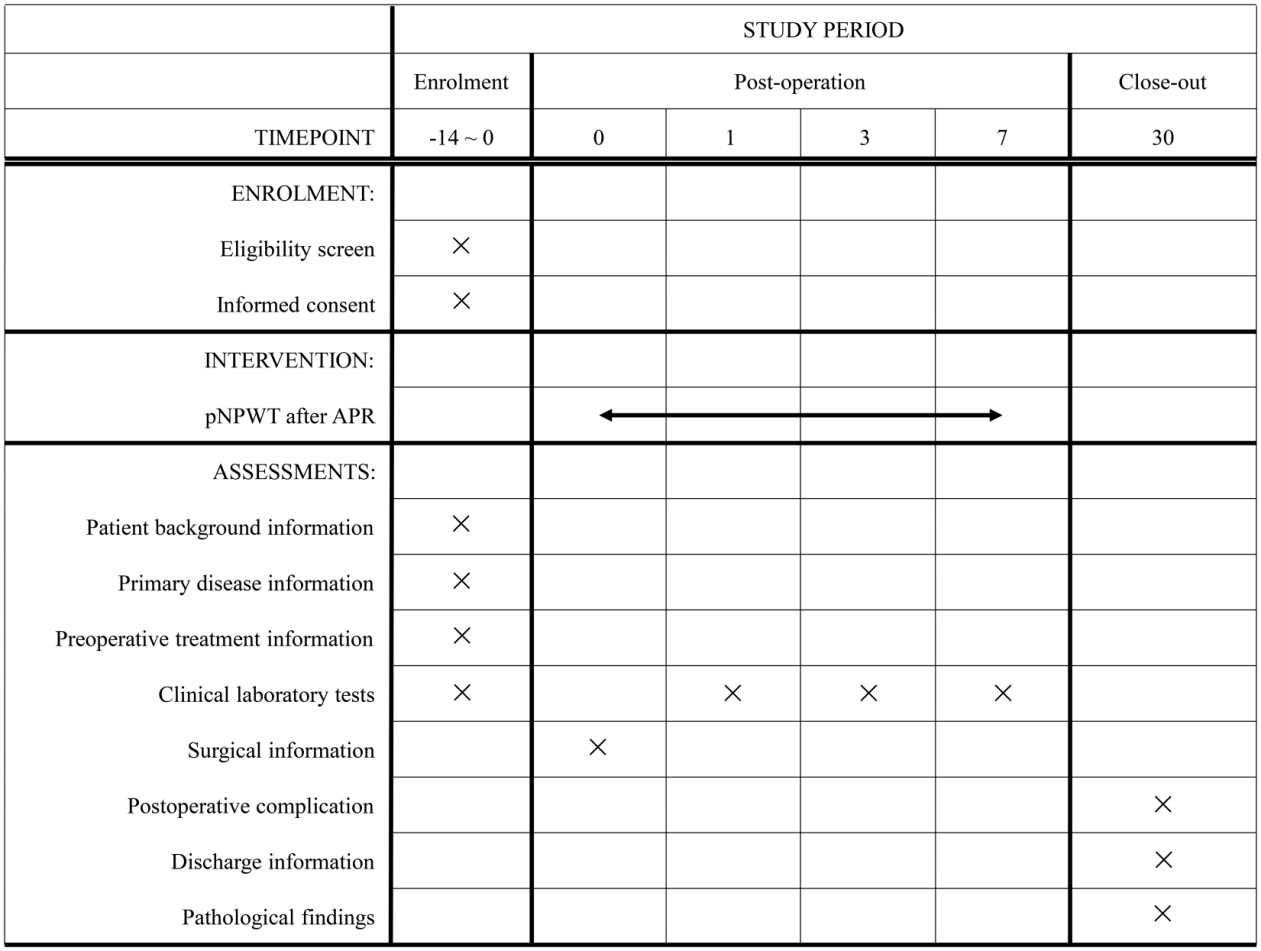
The SPIRIT schedule of VACPAC study. pNPWT: prophylactic negative pressure wound therapy; APR: abdominal perineal resection.

The SPIRIT schedule of this study is shown in Fig 1.

### Intervention

**Fig 2:**
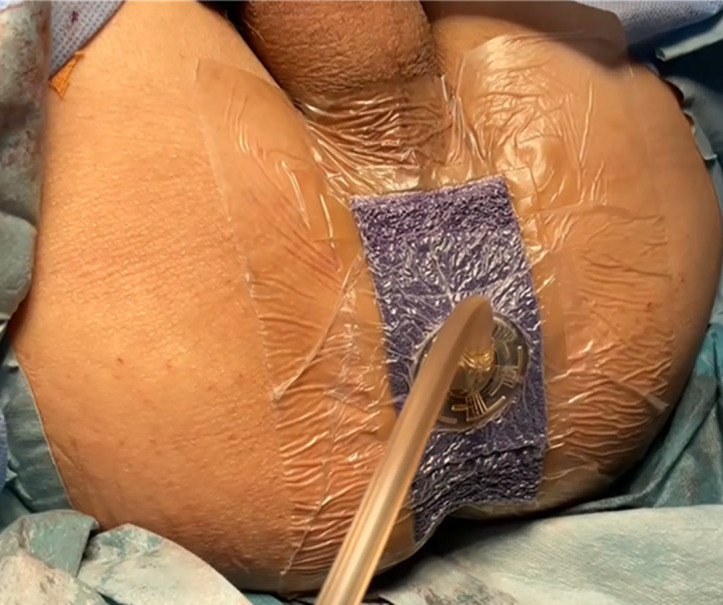
The 3M™ Prevena™ plus customizable system kit on the perineal wound.

The intervention in this study is the application of pNPWT to perineal wounds after APR. Immediately after closure of the perineal skin, the 3M™ Prevena™ Plus Customizable system kit is applied to the perineal wound in the operating room, and pNPWT is continued for up to 7 days, as long as possible (Fig 2). An instructional video demonstrating the application of this kit was provided in advance to each institution. After pNPWT, the investigators perform wound management as usual. If the pNPWT device peels off within 7 days after surgery, the investigator reapplies the device and continues pNPWT until postoperative day 7. However, in cases where investigators intentionally terminate pNPWT due to adverse events, it is not resumed.

### Preoperative management

The investigators use a combination of mechanical and chemical preparations. Mechanical preparation is not specified in the protocol. Chemical preparation is defined as the administration of 1 g of kanamycin and 750 mg of metronidazole twice on the day before surgery. The investigators remove the hair immediately before surgery, instead of shaving.

### Surgery

Disinfectants used for the abdomen and perineum are at the surgeon’s discretion. Surgeons disinfect the perineum after closing the anus. The abdominal procedure is limited to minimally invasive surgeries (laparoscopic or robot-assisted surgery), but for perineal procedures, both open surgery and endoscopic surgery are viable options. The level of central lymph node dissection, the presence or absence of lateral lymph node dissection, autonomic nerve preservation, and the route of sigmoid colon single-hole colostomy are at the surgeon’s discretion.

Immediately before perineal wound closure, the surgeons rinse the area with saline. The method of perineal wound closure (whether to suture subcutaneously or the levator ani muscle, whether to use interrupted skin sutures or buried sutures in the dermis, and what type of suture material to use) is at the surgeon’s discretion.

If the patient does not have an allergy to cefmetazole, it is administered as a prophylactic antibiotic 1 hour before surgery and every 3 hours during surgery. However, the dosage and dosing interval are adjusted as appropriate, depending on the patient’s weight and renal function. If the patient has an allergy to cefmetazole, the choice of antibiotics is determined by each facility. After wound closure, prophylactic antibiotics are not administered [18].

A closed and continuous negative pressure drain is placed transabdominally, but a transperineal drain is not placed. The type of transabdominal drain is at the surgeon’s discretion.

### Postoperative management

If the patient is female, the urinary catheter is kept in place while the pNPWT device is being used to prevent urine contamination of the perineal wound. If perineal SSI occurs, its treatment is at the investigator’s discretion.

### Measurements

We collect patient background information (age, sex, BMI, and comorbidities such as dialysis, inflammatory bowel disease, and diabetes mellitus), primary disease information (TNM classification, tumor location, and histological type), blood test results, and preoperative treatment history (NAC, NACRT, and TNT) as baseline patient information. The precise duration of type 2 diabetes mellitus will not be collected due to the difficulty in accurately determining its onset. Surgical information is collected during surgery, and discharge information is collected at discharge. Postoperative complications and pathology results are collected 30 days after surgery. In addition to these predefined data points, investigators will optionally record free-text comments related to safety and any adverse events.

### Endpoints

The primary endpoint is the incidence of SSI in the perineal wound within 30 days after surgery. SSI includes superficial, deep, and organ/space SSI. The diagnosis of SSI is based on the Centers for Disease Control and Prevention (CDC)/ National Healthcare Safety Network (NHSN) surveillance definition of healthcare-associated infection [19]. The diagnosis of SSI is independently evaluated by two clinicians, at least one of whom is a certified surgeon of the Japan Surgical Society. If the evaluations of the two clinicians differ, the final decision is made in conjunction with the evaluation of a third clinician, who is also a certified surgeon of the Japan Surgical Society.

The secondary endpoints include the incidence proportion of postoperative complications of Clavien–Dindo classification Grade Ⅲ or higher, excluding perineal SSI, postoperative hospital stay, and the completion proportion of pNPWT.

### Follow-up

All patients included in the study are followed for 30 days. Blood tests are performed on the first, third, and seventh days after surgery to assess safety. Adverse events are evaluated throughout the treatment period using the National Cancer Institution Terminology Criteria for Adverse Events version 5.0 (CTCAE v 5.0).

Patients will be discontinued from this clinical study due to protocol deviation if: (i) pNPWT is not actually performed despite study registration. (ii) The patient requests discontinuation of treatment or withdraws consent. (iii) The patient is found to be ineligible after participation. (iv) The principal investigator or sub-investigator deems continued participation unsuitable for safety reasons. Investigators will collect the reasons for discontinuation. If a patient explicitly rejects the use of their data, all collected data from that patient will not be available for analysis.

### Statistical analysis

This study investigates the safety and efficacy of pNPWT on perineal wounds after APR. The safety analysis set (SAS) is defined as the cases that are registered in this study and have had at least part of the pNPWT device applied. The efficacy analysis set (EAS) is defined as cases that are registered in this study, have had at least part of the pNPWT device applied, and have no major study protocol violation.

For perineal wound SSI and other complications, the investigators calculate the incidence proportion and 95% confidence interval for the EAS. The median and interquartile range of the postoperative hospital stay are calculated. Regarding the completion proportion of pNPWT, the investigators calculate the proportion of cases in which pNPWT was applied for 7 days after surgery, along with the 95% confidence interval.

Previous studies have reported that the incidence of perineal wound SSI in cases where pNPWT was applied after APR was 9–15% [14,17]. Therefore, we expect the incidence of SSI in this study to be 12%. Other studies have reported that the incidence of perineal wound SSI in cases where preoperative treatment was performed and pNPWT was not applied after APR was 26–51% [3,4]. Based on this, we set the threshold at 40% for the null hypothesis. The reduction in the incidence of perineal wound SSI from 40% to 12% is clinically meaningful. We will determine that pNPWT is effective if the observed incidence of SSI is statistically significantly lower than 40%. This will be assessed using a two-sided one-sample proportion test. Specifically, a Z-test based on normal approximation will be applied; however, if the conditions for normal approximation are not met (e.g., expected counts of successes or failures are less than 5), an exact binomial test will be used. Efficacy will be concluded if the p-value obtained from this two-sided test is less than 0.05 and the observed SSI incidence is lower than 40%.

In this trial, the sample size for the analysis was calculated as 45 cases with a power of 80%, two-sided alpha level of 5%, and planned recruitment period of one year. Finally, the sample size was set at 50 cases, assuming a 10% rate of discontinuations or exclusions to account for protocol deviations and ineligible cases. This is a multi-institutional trial, and based on the historical number of cases at each participating institution, the anticipated sample size is considered to be achievable. Adjustment for covariates, such as propensity score analysis, will not be performed due to the limited sample size.

This study is not designed in accordance with CDISC standards, and the estimand framework is not applied. Submission to the FDA is not intended, as the study is conducted solely in Japan.

### Interim analysis

An interim analysis is not performed in this study

### Data monitoring

Monitoring is performed to assess study progress, enhance data accuracy, and ensure patient safety. Monitoring officers, appointed by the principal investigator, oversee data management and central monitoring. They have undergone training in the Ethical Guidelines for Medical and Health Research and possess a thorough understanding of the study’s details, Monitoring will be conducted according to a monitoring procedures manual.

### Protection of personal information

In this study, all study personnel must comply with laws on personal information protection. Case report forms will use unique identification codes instead of personally identifiable data, with linkage tables securely stored by the principal investigator. Data management will use identification codes, ensuring participant anonymity in publications. Data collected before consent withdrawal may be used unless complete removal is requested.

### Study status and timeline

Participant recruitment began on 22/01/2025. At the time of submission, participant recruitment, data collection, and outcome analysis have not yet been completed. The estimated date for completion of participant recruitment is 28/02/2026. Data collection is expected to be completed by 31/03/2026, and the results are planned to be published by 31/05/2026.

## Discussion

Especially in high-risk patients for SSI, such as those who have undergone preoperative treatment, the incidence rate of perineal wound SSI after APR is high, and SSI can result in a poor prognosis [3,4,6]. Therefore, the prevention of SSI after APR is important. However, omentoplasty and myocutaneous flap reconstruction for SSI prevention cannot be applied in some cases [9]. On the other hand, pNPWT is easy to apply in most cases without prolonging operative time or invasive procedures. Although pNPWT is expected to reduce SSI by alleviating tension, reducing subcutaneous hematoma and seroma, and creating a sealed environment [13], clear evidence of its efficacy after APR has not yet been established. Therefore, a prospective evaluation of the safety and efficacy of pNPWT on perineal wounds after APR in this study is valuable. To our knowledge, this is the first prospective study evaluating pNPWT after APR, with a particular focus on SSI high-risk patients.

In female patients, applying pNPWT after APR is more challenging than in male patients because the upper edge of the perineal wound is close to the lower edge of the vagina, often leading to air leaks from this area. To prevent air leaks, the 3M™ Prevena™ Plus Customizable system kit is used as the pNPWT device. This system allows customization based on wound shape and can be reapplied as needed. Moreover, we created a simulation video demonstrating the application of this device to perineal wounds after APR to minimize quality differences in pNPWT implementation between institutes. Although previous studies have not reported the completion proportion of pNPWT [14–17], this factor is likely to impact the incidence proportion of SSI after APR. Therefore, we will evaluate the completion proportion of pNPWT as one of the secondary endpoints.

We expect that pNPWT after APR will reduce the incidence of perineal wound SSI. A reduction in SSI may lead to improved prognosis by facilitating the smooth initiation of adjuvant chemotherapy in advanced cancer cases, reducing the costs associated with SSI treatment and prolonged hospital stays, and improving patients’ quality of life after surgery. The results of this study may support a new preventive approach for perineal wound SSI after APR.

## Conclusion

We designed this trial to evaluate the safety and efficacy of pNPWT after APR.

## Supporting information

S1 Checklist Recommended items to address in a clinical trial protocol and related documents. (DOCX)

S1 Protocol Full protocol of the VACPAC study.
(DOCX)
