# Supplementary material for: Protocol for a study to evaluate the safety and efficacy of prophylactic negative pressure wound therapy after abdominal perineal resection (VACPAC study)
Source: PLoS One. 2025 Aug 29;20(8):e0331361. doi: 10.1371/journal.pone.0331361 (PMC12396706; doi:10.1371/journal.pone.0331361)
Supplement: S1 Protocol — Full protocol of the VACPAC study. (DOCX) [file pone.0331361.s002.docx]

VACPAC study

A Multi-Institutional Study on the Safety and Effectiveness of Prophylactic Negative Pressure Wound Therapy for Perineal Wounds

prophylactic Vacuum-Assisted Closure on Perineal wound after Abdominoperineal resection for rectal Cancer

Research Protocol

Principal Investigator: Department of Surgery, Graduate School of Medicine, Kyoto University

Professor, Kazutaka Obama

Version 1.0 January 20, 2025

Revision history

| Version number | Date of creation (revision) |
| --- | --- |
| Version 1.0 | January 20, 2025 |

Ethical Principles

All personnel involved in this study shall comply with the "Clinical Trials Act (Act No. 16 of 2017)" (promulgated on April 14, 2017) and the "Enforcement Regulations on the Clinical Trials Act (Order of the Ministry of Health, Labour and Welfare No. 17 of 2018)" (promulgated on February 28, 2018), as well as other relevant legal regulations. Additionally, they shall adhere to applicable ethical guidelines, including the "World Medical Association Declaration of Helsinki" (revised in October 2013).

Overview

| Objective | | To prospectively evaluate the safety and effectiveness of prophylactic negative pressure wound therapy (pNPWT) for perineal closure wounds following abdominoperineal resection for rectal malignancies. |
| --- | --- | --- |
| Study Phase | | Phase 2 |
| Planned Start Date for Patient Enrollment | | December 1, 2024 |
| Study Duration | | From the date of public disclosure of the study plan to May 31, 2026 |
| Planned Number of Participants | | 50 cases |
| Study Type | | Interventional study |
| Study Design | | Single arm study  Open (masking not used)  Uncontrolled  Single assignment  Prevention purpose |
| Coverage of Costs | | No additional medical expense burden on participants |
| Countries of Recruitment | | Only Japan |
| Key  Inclusion & Exclusion Criteria | Inclusion Criteria | 1) Patients undergoing abdominoperineal resection (APR) for rectal malignancies.  2) Patients meeting the insurance coverage criteria for the PREVENA incision management system, specifically:  (a) Patients who underwent neoadjuvant therapy (chemotherapy, chemoradiotherapy, or both).  (b) Obese patients with a BMI ≥ 30.  (c) Diabetic patients with HbA1c (NGSP) ≥ 7.0%.  (d) Patients receiving steroid therapy.  (e) Patients undergoing chronic maintenance dialysis.  (f) Patients with malnutrition (meeting one of the following criteria):  3) Patients aged 18 years or older, regardless of gender, with an ECOG Performance Status (PS) of 0 or 1.  4) Obtain written consent to participate in the study |
|  | Exclusion Criteria | 1) Emergency surgery cases.  2) Cases with obviously contaminated/infected wounds where primary closure is not advisable.  3) Myocutaneous flap reconstruction is scheduled to be performed.  4) Patients with allergies or hypersensitivity to silver or acrylic adhesives.  5) Other cases deemed inappropriate for study participation by the principal or sub-investigators |
|  | Minimum Age | 18 years |
|  | Maximum Age | None |
|  | Gender | Both male and female participants are eligible |
| Discontinuation Criteria | | 1) The participant was registered but did not receive prophylactic negative pressure wound therapy (pNPWT).  2) The participant requests discontinuation of treatment or withdrawal of consent.  3) The participant is found to be ineligible after the start of treatment.  4) The principal or sub-investigator determines that continuation of the study is difficult for safety or other reasons. |
| Target Disease | | Rectal malignancies |
| Intervention Details | | Following primary suture closure of the perineal wound after APR, prophylactic negative pressure wound therapy (pNPWT) using the Prevena Plus Customizable Kit will be applied for one week. |
| Primary Outcome(s) | | Incidence rate of surgical site infection (SSI) in the perineal wound within 30 days postoperatively. |
| Secondary Outcome(s) | | Incidence of postoperative complications other than perineal SSI.  Postoperative hospital stay duration.  Completion rate of pNPWT. |

Shema


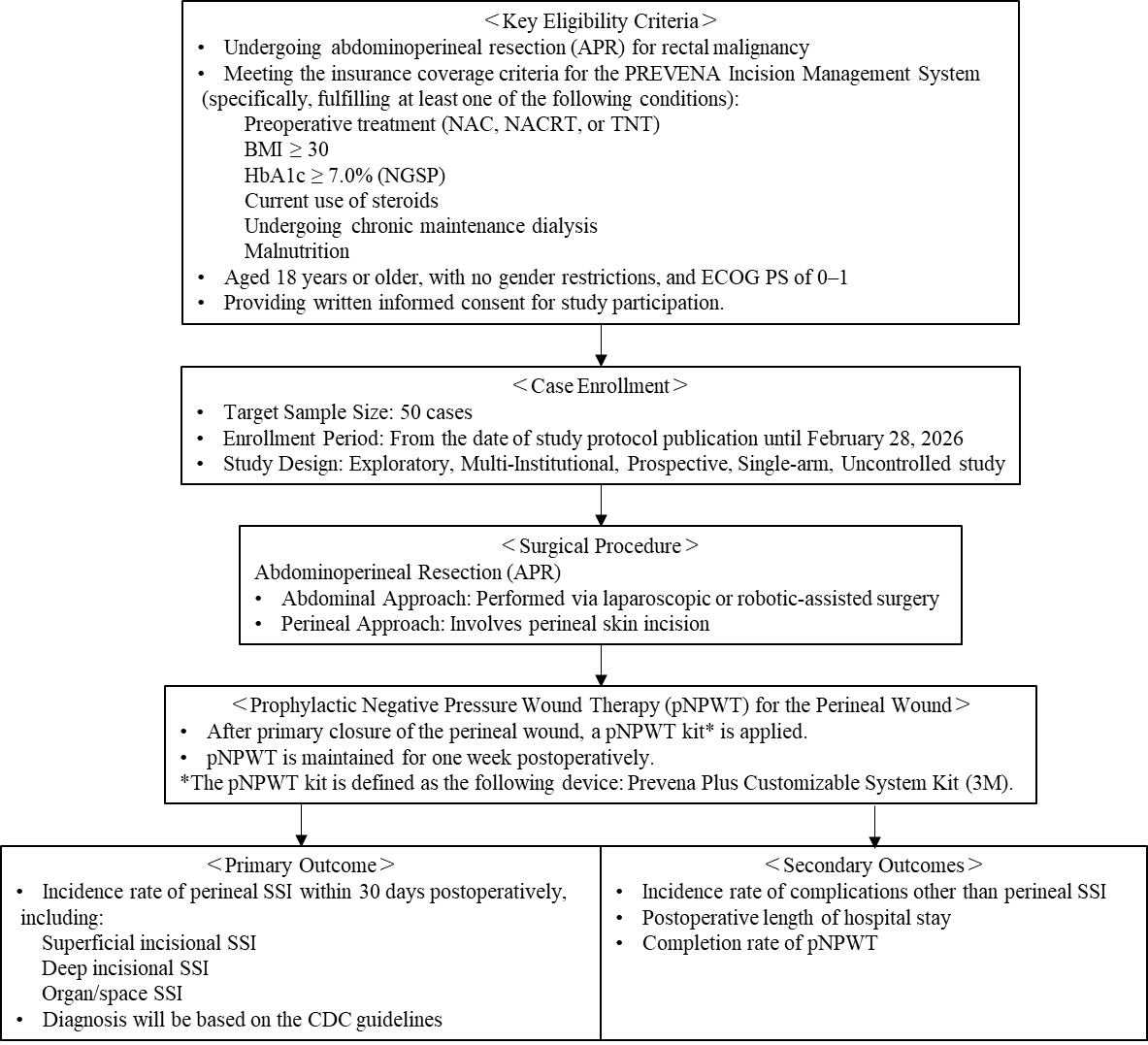


Table of Contents

[1. Background and Significance 9](#_Toc191825328)

[1.1. Target Disease and Previous Studies Related to This Research 9](#_Toc191825329)

[1.2. Positioning and Significance of This Study and the Investigational Device 9](#_Toc191825330)

[1.3. Development of This Study Plan 10](#_Toc191825331)

[2. Objective 10](#_Toc191825332)

[3. Study Subjects and Target Disease 10](#_Toc191825333)

[4. Eligibility Criteria 10](#_Toc191825334)

[4.1. Inclusion Criteria 10](#_Toc191825335)

[4.2. Rationale for Inclusion Criteria 11](#_Toc191825336)

[4.3. Exclusion Criteria 11](#_Toc191825337)

[4.4. Rationale for Exclusion Criteria 11](#_Toc191825338)

[5. Ethical Considerations 11](#_Toc191825339)

[5.1. Informed Consent Process 11](#_Toc191825340)

[5.2. Preparation and Revision of the Informed Consent Form 12](#_Toc191825341)

[5.3. Protection of Personal Information 12](#_Toc191825342)

[5.4. Compensation for Health Damage 12](#_Toc191825343)

[5.5. Handling of Incidental Findings 13](#_Toc191825344)

[5.6. Handling of Participant Inquiries and Complaints 13](#_Toc191825345)

[6. Study Design 13](#_Toc191825346)

[6.1. Overview of Study Design 13](#_Toc191825347)

[6.2. Methods 13](#_Toc191825348)

[6.2.1. Implementation of pNPWT and Treatment Duration 13](#_Toc191825349)

[6.2.2. Standardized Procedures Across Institutions 14](#_Toc191825350)

[6.2.3. Prohibited Concomitant Medications 15](#_Toc191825351)

[6.2.4. Management of SSI After Occurrence 15](#_Toc191825352)

[6.2.5. Surgical Considerations 15](#_Toc191825353)

[6.3. Discontinuation Criteria 15](#_Toc191825354)

[6.4. Post-Study Treatment 16](#_Toc191825355)

[7. Investigational Device 16](#_Toc191825356)

[7.1. Overview of the Investigational Device 16](#_Toc191825357)

[7.2. Device Classification 16](#_Toc191825358)

[7.3. Distribution, Storage, Management, and Return of the Device 16](#_Toc191825359)

[7.4. Dentification and Prevention of Misuse 16](#_Toc191825360)

[8. Study Procedures 16](#_Toc191825361)

[8.1. Patient Enrollment Procedures 16](#_Toc191825362)

[8.2. Observation, Examination, and Reporting Items 17](#_Toc191825363)

[8.2.1. Baseline (Preoperative) Patient Information 17](#_Toc191825364)

[8.2.2. Surgical and Postoperative Information 17](#_Toc191825365)

[8.3. Study Schedule 18](#_Toc191825366)

[9. Evaluation Criteria 18](#_Toc191825367)

[9.1. Primary Outcome 18](#_Toc191825368)

[9.2. Secondary Outcomes 19](#_Toc191825369)

[9.3. Criteria for Diagnosing SSI 19](#_Toc191825370)

[9.3.1. Superficial incisional SSI 19](#_Toc191825371)

[9.3.2. Deep incisional SSI 19](#_Toc191825372)

[9.3.3. Organ/Space SSI 20](#_Toc191825373)

[10. Adverse Events and Study-Related Conditions 20](#_Toc191825374)

[10.1. Definition and Management of Adverse Events and Study-Related Conditions 20](#_Toc191825375)

[10.2. Definition and Management of Serious Adverse Events and Study-Related Conditions 21](#_Toc191825376)

[10.3. Management of Device Malfunctions 21](#_Toc191825377)

[10.4. Expected Adverse Events 21](#_Toc191825378)

[11. Expected Benefits and Risks/Discomfort Associated with Study Participation 21](#_Toc191825379)

[11.1. Expected Benefits 21](#_Toc191825380)

[11.2. Burdens and Potential Risks 22](#_Toc191825381)

[11.3. Measures to Minimize Burdens and Risks 22](#_Toc191825382)

[12. Statistical Analysis 22](#_Toc191825383)

[12.1. Analysis Population 22](#_Toc191825384)

[12.1.1. Safety Analysis Set (SAS) 22](#_Toc191825385)

[12.1.2. Efficacy Analysis Set (EAS) 22](#_Toc191825386)

[12.2. Analysis Methods 22](#_Toc191825387)

[12.2.1. Primary Analysis 22](#_Toc191825388)

[12.2.2. Secondary Analyses 22](#_Toc191825389)

[12.3. Target Sample Size and Rationale for Determination 23](#_Toc191825390)

[12.4. Changes to the Statistical Analysis Plan 23](#_Toc191825391)

[13. Data Management 23](#_Toc191825392)

[13.1. Case Report Form (CRF) Completion and Submission 23](#_Toc191825393)

[13.2. Handling of Data 24](#_Toc191825394)

[13.3. Identification of Source Documents 24](#_Toc191825395)

[14. Direct Access to Source Documents 24](#_Toc191825396)

[15. Monitoring and Auditing 24](#_Toc191825397)

[15.1. Monitoring 24](#_Toc191825398)

[15.2. Designation of Monitors 24](#_Toc191825399)

[15.3. Monitoring Procedures 25](#_Toc191825400)

[15.4. Reporting of Monitoring Results 25](#_Toc191825401)

[15.5. Auditing 25](#_Toc191825402)

[16. Approval and Amendments of the Study Protocol 25](#_Toc191825403)

[16.1. Approval, Modification, and Revision of the Study Protocol 25](#_Toc191825404)

[16.2. Deviations from the Study Protocol 25](#_Toc191825405)

[17. Study Completion, Early Termination, Suspension, and Resumption 26](#_Toc191825406)

[17.1. Study Completion 26](#_Toc191825407)

[17.2. Early Termination, Suspension, and Resumption of the Study 26](#_Toc191825408)

[18. Reporting to Institutional Administrators of Participating Medical Institutions 27](#_Toc191825409)

[19. Study Funding and Financial Considerations 27](#_Toc191825410)

[19.1. Funding Sources and Conflicts of Interest 27](#_Toc191825411)

[19.2. Research-Related Costs, Participant Burden, and Compensation 27](#_Toc191825412)

[20. Handling of Samples and Data After Study Completion 27](#_Toc191825413)

[21. Attribution of Research Outcomes and Publication of Results 28](#_Toc191825414)

[21.1. Attribution of Research Outcomes 28](#_Toc191825415)

[21.2. Method of Public Disclosure of Research Information 28](#_Toc191825416)

[22. Study Implementation Period 28](#_Toc191825417)

[23. Study Organization 28](#_Toc191825418)

[24. Certified Clinical Research Review Board 32](#_Toc191825419)

[25. References 32](#_Toc191825420)

[26. Appendix 33](#_Toc191825421)

# Background and Significance

## Target Disease and Previous Studies Related to This Research

The incidence of surgical site infection (SSI) following abdominoperineal resection (APR) for rectal malignancies is high ^(1)(2)^. Among them, perineal wound SSI accounts for 30–64% ^(3)(4)^. Once an SSI occurs, it may lead to an extended hospital stay, increased medical costs, and a decline in the patient’s quality of life ^(5)^. Furthermore, rectal cancer patients requiring APR often have advanced-stage disease and are candidates for adjuvant chemotherapy postoperatively. A delay in initiating chemotherapy due to perineal wound SSI may negatively impact long-term prognosis ^(5)^. Therefore, preventing perineal wound SSI is a critical issue.

Several factors contribute to SSI after APR. Perineal wounds experience significant tension during ambulation, which may lead to wound dehiscence ^(5)^. Additionally, blood and exudates accumulating in the pelvic dead space after organ resection may serve as a source of infection, leading to pelvic abscesses and wound infections ^(4)^. Patients undergoing neoadjuvant therapy, such as neoadjuvant chemotherapy (NAC), neoadjuvant chemoradiotherapy (NACRT), or total neoadjuvant therapy (TNT), may experience delayed wound healing due to tissue damage and immunosuppression ^(6)^.

Various preventive measures for perineal wound SSI have been reported, including omentoplasty and pelvic floor reconstruction using myocutaneous flaps such as the rectus abdominis or gracilis muscle ^(7)(8)(9)^. However, perineal wound SSI remains common even with these approaches. Additionally, myocutaneous flaps require plastic surgical techniques and carry risks such as flap necrosis ^(10)^ and prolonged operative time ^(4)^. Omentoplasty may not be feasible in cases involving inflammation or prior omentectomy ^(11)^. Given these limitations, a novel approach to reduce perineal wound SSI is necessary.

## Positioning and Significance of This Study and the Investigational Device

Negative pressure wound therapy (NPWT), also known as vacuum-assisted closure (VAC), is a non-invasive method that promotes wound healing by applying negative pressure in a closed environment ^(12)^. NPWT has shown favorable outcomes in treating chronic and complex wounds, such as diabetic foot gangrene and mediastinitis after cardiac surgery ^(13)^. In gastrointestinal surgery, NPWT has been effective in managing open midline wounds following peritonitis and perineal open wounds after APR ^(14)^.

Recently, prophylactic NPWT (pNPWT) has gained attention for use in patients at high risk of wound complications. The 2018 Perioperative Management Guidelines for SSI Prevention in Gastrointestinal Surgery by the Japan Society for Surgical Infection suggest that pNPWT may reduce incisional SSI in gastrointestinal surgery ^(15)^. The proposed mechanisms of pNPWT include reducing wound tension by approximating wound edges, decreasing hematoma and seroma formation in the subcutaneous space, and creating a sealed environment that protects against external contamination. These combined effects are believed to lower the risk of SSI ^(16)(17)^. Observational studies and randomized controlled trials (RCTs) have been conducted in gastrointestinal surgery for non-infected closed wounds ^(18)(19)^. However, there are few reports specifically on pNPWT for perineal wounds after APR. Some studies suggest that pNPWT may reduce perineal wound SSI rates by 25–30% ^(20)^, but these studies have small sample sizes and lack definitive evidence.

## Development of This Study Plan

In March 2021, the Prevena Incision Management System (IMS) (3M, St. Paul, Minnesota) became the first pNPWT device approved in Japan. Prior to this clinical trial, a preliminary study was conducted at Fukuoka University Hospital, where Prevena was applied to perineal closed wounds after APR. In a control group of 117 patients who underwent primary closure alone without pNPWT, perineal wound SSI was observed in 24%. In contrast, in the pNPWT group (10 patients), no cases of perineal wound SSI were reported.

This study aims to prospectively evaluate the safety and effectiveness of pNPWT for perineal closed wounds after APR, thereby clarifying its effectiveness, indications, and clinical significance. Since April 2022, pNPWT has been covered by the Japanese insurance system, and its use for SSI prevention in high-risk patients may become the standard of care. The data obtained in this study will provide the basis for a future large-scale trial in gastrointestinal surgery, further establishing the efficacy and safety of pNPWT for perineal and other surgical wounds.

# Objective

This study aims to prospectively evaluate the safety and effectiveness of prophylactic negative pressure wound therapy (pNPWT) for perineal closed wounds after abdominoperineal resection (APR) in high-risk patients for surgical site infections (SSI).

# Study Subjects and Target Disease

Patients who have undergone APR for malignant rectal tumors.

# Eligibility Criteria

Study subjects will be enrolled only if they meet all the inclusion criteria and do not fall under any of the exclusion criteria.

## Inclusion Criteria

##### Patients undergoing APR* for malignant rectal tumors (*APR is defined as rectal amputation involving a perineal skin incision).

##### Patients who meet the insurance criteria for the PREVENA Incision Management System by satisfying at least one of the following conditions:

###### Undergoing preoperative treatment (chemotherapy or chemoradiotherapy)

###### BMI ≥ 30

###### Diabetic patients with hemoglobin A1c (NGSP) ≥ 7.0%

###### Receiving steroid therapy

###### Undergoing chronic maintenance dialysis

###### In a state of malnutrition, as indicated by one of the following:

1. Unintentional weight loss > 5% within 6 months or > 10% over 6 months
2. BMI < 18.5 for patients under 70 years old, or BMI < 20 for those 70 years and older
3. Calf circumference < 34 cm for men or < 33 cm for women, or skeletal muscle mass < 7.0 kg/m² (men) and < 5.4 kg/m² (women) based on dual-energy X-ray absorptiometry (DXA) or < 7.0 kg/m² (men) and < 5.7 kg/m² (women) based on bioelectrical impedance analysis (BIA)

##### Patients aged 18 years or older with an Eastern Cooperative Oncology Group (ECOG) Performance Status (PS) of 0 or 1

##### Patients who have provided written informed consent for study participation

## Rationale for Inclusion Criteria

Criteria (1) and (2) define the target patient population for this study.

Criterion (3) is set to ensure patient safety.

Criterion (4) is required for ethical study conduct.

## Exclusion Criteria

1. Patients undergoing emergency surgery
2. Patients with a significantly contaminated or infected wound where primary closure is not advisable
3. Patients scheduled to undergo myocutaneous flap reconstruction
4. Patients with known allergies or hypersensitivity to silver or acrylic-based adhesives
5. Any other patients deemed unsuitable for safe study participation by the principal investigator or sub-investigators

## Rationale for Exclusion Criteria

Criteria (1) – (3) are set to minimize factors that could affect the study’s efficacy evaluation.

Criteria (4) – (5) are established for safety considerations.

# Ethical Considerations

## Informed Consent Process

The principal investigator or sub-investigators will provide a thorough explanation of the study to potential participants before obtaining consent. The informed consent documents must be reviewed and approved by the Certified Clinical Research Review Board (CRB) and the hospital director before use. Participants will be given sufficient time to review the study details and ask questions. Written informed consent will be obtained from all participants before study enrollment.

The investigator will ensure that the informed consent form includes the following:

・The name of the physician providing the explanation and the date of explanation.

・The participant’s name and the date of consent.

・A copy of the signed consent form will be provided to the participant.

・The original consent form will be securely stored at the medical institution conducting the study.

If new information arises that may impact the participant’s decision to continue in the study, the investigator will promptly inform the participant. If the consent form is revised, participants will be re-consented using the updated form.

## Preparation and Revision of the Informed Consent Form

The principal investigator is responsible for preparing and revising the informed consent form as needed. Any revisions must be approved by the CRB and the hospital director before use. The consent form must comply with applicable regulations and must not include misleading information.

If new safety or investigational device-related information becomes available that could impact the participant’s willingness to continue, the consent form will be updated and reapproved by the relevant authorities.

## Protection of Personal Information

All study personnel must adhere to applicable laws and regulations regarding personal information protection. Personal information obtained during the study must not be disclosed without justification, even after study completion.

Case report forms will not include personally identifiable information such as names, addresses, or phone numbers. Instead, unique participant identification codes will be used. The correspondence table linking these codes with personal data will be securely stored by the principal investigator.

Study data management personnel will use identification codes or registration numbers to handle data. When publishing study results, every effort will be made to ensure that participants are not personally identifiable.

Data collected before withdrawal of consent may still be used unless the participant explicitly requests complete removal of their data.

## Compensation for Health Damage

If a participant experiences an adverse event related to the study, they will receive appropriate medical care. However, treatment costs will be covered under their health insurance, and no additional financial compensation will be provided. The study does not include clinical research insurance coverage.

## Handling of Incidental Findings

Although this study is not expected to yield significant genetic or health-related findings, any incidental findings relevant to a participant’s health will be communicated to the treating physician or, in the case of healthy volunteers, participants will be advised to seek medical attention.

## Handling of Participant Inquiries and Complaints

The study team will provide a designated contact point for participant inquiries, complaints, or concerns. This contact information will be included in the informed consent documents. The responsible personnel will respond promptly and appropriately.

・Contact Information

・Takuya Takami:

Department of Surgery, Graduate School of Medicine, Kyoto University, Graduate Student

Address: 54 Shogoin Kawaharacho, Sakyo-ku, Kyoto-shi, Kyoto

Tel: 075-751-3111

E-mail: t_takami@kuhp.kyoto-u.ac.jp

・Yoshiro Itatani:

Department of Surgery, Graduate School of Medicine, Kyoto University, Associate Professor

Address: 54 Shogoin Kawaharacho, Sakyo-ku, Kyoto-shi, Kyoto

Tel: 075-751-3111

E-mail: itatani@kuhp.kyoto-u.ac.jp

・Kyoto University Hospital Clinical Research Consultation Desk

Tel: 075-751-4748

Email: [ctsodan@kuhp.kyoto-u.ac.jp](mailto:ctsodan@kuhp.kyoto-u.ac.jp)

# Study Design

## Overview of Study Design

This study is a multicenter, single-arm, open-label, non-controlled interventional trial.

## Methods

### Implementation of pNPWT and Treatment Duration

Immediately after perineal wound closure in two or three layers (skin, subcutaneous tissue, and/or levator ani muscle) following APR, the pNPWT device (Prevena Plus Customizable Kit) will be applied to the perineal wound in the operating room. Continuous negative pressure will be applied for 7 days postoperatively. After 7 days, the device will be removed, and routine wound management will be performed. If the pNPWT device detaches within the first 7 days postoperatively, an attempt will be made to reapply it and continue treatment until day 7. If the device is intentionally removed due to adverse events, reapplication will not be performed.

### Standardized Procedures Across Institutions

1. Bowel Preparation

Mechanical and chemical bowel preparation (laxatives and oral antibiotics) will be used. The choice of laxative and administration method is left to the discretion of each institution.

Oral antibiotics (kanamycin 1 g and metronidazole 750 mg) will be administered twice on the day before surgery.

1. Preoperative Hair Removal

No shaving will be performed before surgery; hair removal will be done immediately before the operation.

1. Surgical Site Disinfection

The type of disinfectant used for abdominal preparation is not specified (left to institutional policy). For perineal disinfection, the anus will be temporarily closed before applying disinfectant. The choice of disinfectant for the perineal region is also left to institutional policy.

1. Surgical Approach for APR

Abdominal approach: Either laparoscopic or robotic-assisted surgery

Perineal approach: Both open and endoscopic approaches are acceptable

1. Perineal Wound Closure

The method of perineal wound closure is not specified (left to institutional policy). A typical closure method includes:

Two or three-layer closure involving skin, subcutaneous tissue, and/or levator ani muscle.

Subcutaneous closure: Monofilament absorbable sutures with interrupted or continuous suturing.

Skin closure: Monofilament non-absorbable interrupted sutures (removed after wound healing is deemed complete) or monofilament absorbable subcutaneous sutures.

1. Prophylactic Intravenous Antibiotics

Preoperative: Cefmetazole will be administered within 1 hour before surgery. If the patient has an allergy to cefmetazole, an alternative antibiotic will be selected at the discretion of each institution.

Intraoperative: If using cefmetazole, the same antibiotic will be administered every 3 hours from the initial dose. The dosage and intervals may be adjusted based on renal function and body weight.

Postoperative: No prophylactic intravenous antibiotics will be given after wound closure.

1. Other Measures

Perineal wounds will be irrigated with saline before closure.

A closed suction drain will be placed in the pelvic floor via an abdominal approach.

No perineal drain will be used.

In female patients, a urinary catheter will be maintained for the first postoperative week to prevent perineal wound contamination.

If subcutaneous sutures are used for skin closure, steri-strips will be applied after pNPWT removal to approximate the wound edges.

### Prohibited Concomitant Medications

There are no prohibited concomitant medications.

### Management of SSI After Occurrence

The treatment approach for perineal wound SSI is not predefined (left to institutional policy). No restrictions are placed on additional treatment after completion or discontinuation of the study intervention.

### Surgical Considerations

No restrictions are placed on the extent of lymph node dissection, preservation of autonomic nerves, or the approach to sigmoid colostomy. The surgical approach will be laparoscopic or robotic-assisted, and open surgery is excluded.

## Discontinuation Criteria

A participant’s continuation in the study may be discontinued under the following conditions:

1. The participant is enrolled but does not receive pNPWT postoperatively.
2. The participant requests withdrawal of treatment or consent is revoked.
3. It is determined after treatment initiation that the participant does not meet the eligibility criteria.
4. The principal investigator or sub-investigators deem that continuing the study poses safety concerns.

If a participant withdraws consent, the investigator will confirm whether the data collected up to that point may still be used in the study. If the participant refuses all data use, the study office will be informed accordingly.

## Post-Study Treatment

No restrictions are placed on treatments administered after study completion or early termination (left to institutional policy).

# Investigational Device

## Overview of the Investigational Device

The pNPWT device used in this study is the:

・3M™ Prevena Plus Customizable Kit

## Device Classification

Class III (under Japan’s medical device classification system).

## Distribution, Storage, Management, and Return of the Device

Since this device is an approved medical device, it is routinely available in the operating rooms of participating institutions. During the study period, it will be managed and stored in the same manner as in regular clinical practice.

The principal investigator will confirm that each participating institution has appropriate protocols for the storage, management, and use of the device.

## Dentification and Prevention of Misuse

Since this device is also used in standard clinical practice, no special labeling or additional identification measures will be implemented.

# Study Procedures

## Patient Enrollment Procedures

After obtaining informed consent, the principal investigator or sub-investigators will assess eligibility based on the criteria outlined in 8.2. Observation, Examination, and Reporting Items. If eligibility is confirmed, patient enrollment will be recorded in an electronic data capture system (REDCap).

Contact Information for Enrollment Inquiries:

・Kyoto University Hospital, Department of Surgery

・Address: 54 Shogoin Kawaharacho, Sakyo-ku, Kyoto 606-8507, Japan

・Phone: +81-75-366-7595 (Weekdays, 9 AM – 4 PM)

・Email: t_takami@kuhp.kyoto-u.ac.jp

## Observation, Examination, and Reporting Items

### Baseline (Preoperative) Patient Information

Eligibility will be confirmed based on the following baseline information.

Clinical tests performed within 14 days before surgery will be considered valid.

1. Patient background information

Date of birth, date of consent, age at consent, sex.

1. Physical examination

Height, weight, BMI, ECOG-PS.

1. Primary disease information

TNM classification (clinical stage), tumor location, histological type (including carcinoma, GIST, and NET). (Following Japanese Classification of Colorectal, Appendiceal, and Anal Carcinoma, 9th Edition ^(22)^.)

1. Laboratory tests

WBC, Neu, Hb, PLT, T-Bil, AST, ALT, LDH, ALB, CRP, CRE, eGFR, CCr, Na, K, Cl, HbA1c (NGSP)

1. Smoking status (Brinkman index)
2. Presence of chronic dialysis
3. Use of steroids
4. Comorbid inflammatory bowel disease (IBD)
5. Diabetes mellitus
6. Preoperative treatment history

NAC、NACRT、TNT

### Surgical and Postoperative Information

Clinical tests will be conducted on postoperative day 1 (±1 day allowed), day 3 (±1 day allowed), and day 7 (±2 days allowed).

Surgical information, hospitalization details, surgical outcomes, and pathology results will be recorded as needed.

1. Surgical Information

Date of surgery, duration of surgery, type of disinfectant used, estimated blood loss, presence of blood transfusion, type of antibiotics used.

Abdominal approach: Laparoscopic, robotic-assisted.

Perineal approach: Open, endoscopic.

Skin closure method (interrupted sutures, subcutaneous buried sutures) and type of suture used.

Amount of saline used for wound irrigation.

Lateral lymph node dissection (LLND): Presence or absence (if present, specify left or right).

1. Laboratory Tests

WBC, CRP, other abnormal values (CTCAE Grade ≥2).

1. Hospitalization and Discharge Information

Date of admission, date of discharge or transfer.

1. Surgical Outcomes

Postoperative complications (perineal wound SSI and other complications).

Whether pNPWT was maintained for the full 7-day period.

If pNPWT was removed prematurely, specify on which postoperative day.

1. Pathology Results

ypTNM classification, surgical margin status, assessment of response to chemotherapy.

(Following Japanese Classification of Colorectal, Appendiceal, and Anal Carcinoma, 9th Edition ^(22^).

## Study Schedule

The study schedule is outlined in the table below. Routine postoperative clinical and imaging examinations may be performed as needed, regardless of this schedule.


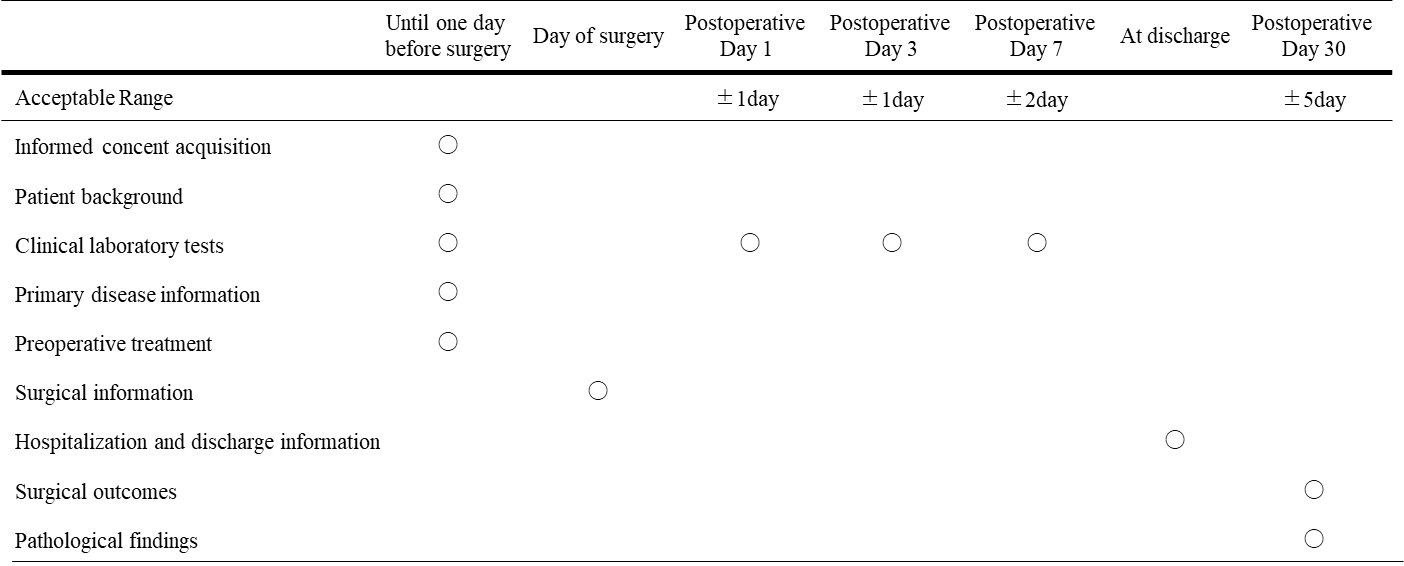


# Evaluation Criteria

## Primary Outcome

1. Incidence of perineal wound SSI

The proportion of patients who develop perineal wound SSI from the time of enrollment until 30 days postoperatively. SSI includes superficial incisional SSI, deep incisional SSI, and organ/space SSI. If a single patient develops multiple SSIs (e.g., both superficial incisional SSI and organ/space SSI), they will be counted as one case.

Diagnosis of SSI will follow the definitions published by the Centers for Disease Control and Prevention (CDC) in the National Healthcare Safety Network (NHSN) criteria ^(21)^.

## Secondary Outcomes

1. Incidence of postoperative complications other than perineal wound SSI

The proportion of patients who experience complications classified as Grade III or higher on the Clavien-Dindo classification scale.

1. Postoperative length of hospital stay

The duration from the date of surgery to discharge or transfer.

1. Completion rate of pNPWT

The proportion of patients in the pNPWT group who successfully maintained pNPWT application for 7 days postoperatively.

## Criteria for Diagnosing SSI

SSI's diagnostic criteria follow the CDC's published definition ^(21^).

SSI will be diagnosed according to the definitions published by the CDC ^(21)^. A checklist will be used to confirm the diagnosis based on signs and symptoms. Two clinicians (at least one of whom must be a board-certified surgeon) will independently evaluate the case. If their assessments differ, a third board-certified surgeon will be consulted to make the final determination.

The wound dehiscence with signs of infection will be classified as SSI. The wound dehiscence without signs of infection will be classified as a postoperative complication other than SSI.

### Superficial incisional SSI

A case will be classified as superficial incisional SSI if all of the following conditions are met:

1. The infection occurs within 30 days postoperatively.
2. The infection is limited to the skin and subcutaneous tissue.
3. At least one of the following criteria is met:
4. Purulent discharge from the superficial incision.
5. Organisms isolated from aseptically obtained fluid or tissue from the superficial incision.
6. The superficial incision is deliberately opened by the surgeon, with or without positive culture, and at least one of the following symptoms is present: pain, tenderness, localized swelling, erythema, or heat. A negative culture result does not meet this criterion.
7. The attending physician or surgeon diagnoses the condition as superficial incisional SSI.

### Deep incisional SSI

A case will be classified as deep incisional SSI if all of the following conditions are met:

1. The infection occurs within 30 days postoperatively.
2. The infection involves deep soft tissues, such as fascia and muscle layers.
3. At least one of the following criteria is met:
4. Purulent discharge from the deep incision, excluding the organ/space.
5. The deep incision spontaneously dehisces or is deliberately opened by the surgeon, with or without positive culture, and at least one of the following symptoms is present: fever (>38°C), localized pain, or tenderness. A negative culture result does not meet this criterion.
6. An abscess or other evidence of infection involving the deep incision is detected via direct examination, reoperation, histopathology, or radiologic imaging.
7. The attending physician or surgeon diagnoses the condition as deep incisional SSI.

### Organ/Space SSI

A case will be classified as organ/space SSI if all of the following conditions are met:

1. The infection occurs within 30 days postoperatively and is deemed to be related to the surgical procedure.
2. The infection involves any part of the body that was manipulated during the surgery, excluding the incision, fascia, or muscle layers.
3. At least one of the following criteria is met:
4. Purulent discharge from a drain placed in the organ/space through a stab wound.

Organisms isolated from aseptically obtained fluid or tissue samples from the organ/space.

1. An abscess or other evidence of infection involving the organ/space is detected via direct examination, reoperation, histopathology, or radiologic imaging.
2. The attending physician or surgeon diagnoses the condition as organ/space SSI.

# Adverse Events and Study-Related Conditions

## Definition and Management of Adverse Events and Study-Related Conditions

An adverse event (AE) is defined as any unfavorable or unintended disease, injury, or medical condition that occurs in a study subject, regardless of its causal relationship with the study. Under the Japanese Clinical Trials Act, "study-related conditions" refer to adverse events where a causal relationship with the study intervention cannot be ruled out.

Adverse events occurring from the start of the study until the end of the observation period (30 days postoperatively or the later of discharge/transfer) will be reported. If an adverse event meeting the definition of a study-related condition occurs after the observation period, it will also be reported.

If an adverse event occurs, the principal investigator or sub-investigators must take appropriate measures and monitor the patient’s progress. Details of adverse event management are outlined in the Procedures for Handling Study-Related Conditions (see Appendix).

## Definition and Management of Serious Adverse Events and Study-Related Conditions

Serious adverse events include any of the following:

1. Death
2. Life-threatening events
3. Permanent disability
4. Events that may result in permanent disability
5. Hospitalization or prolonged hospitalization due to treatment
6. Other events deemed serious
7. Congenital anomalies in future generations.

If a serious adverse event occurs, the principal investigator or sub-investigators must take appropriate measures and follow the "Procedures for Handling Study-Related Conditions." The event must be reported immediately to the CRB, institutional administrators, and the lead investigator.

If the event is deemed a study-related condition, the lead investigator will report it to the Minister of Health, Labour and Welfare (via the Pharmaceuticals and Medical Devices Agency), institutional investigators, and the device provider in accordance with the Japanese Clinical Trials Act.

## Management of Device Malfunctions

If a malfunction occurs in the study device, the principal investigator or sub-investigator must report it to the institutional principal investigator. The institutional principal investigator must notify the institutional administrator and the lead investigator. If the malfunction is deemed to pose a risk of a serious adverse event, the lead investigator will report it to the CRB within 30 days of becoming aware of the issue. If a serious adverse event occurs due to the malfunction, management will follow the procedures outlined in 10.2.

## Expected Adverse Events

According to the latest package insert for the 3M™ Prevena Plus Customizable Kit, serious adverse events such as bleeding and infection have been reported. Device malfunctions include fire and false alarms. Other adverse events include allergic reactions, maceration, lymphatic leakage, epidermal necrosis, delayed or worsened wound healing, soft tissue damage, contact dermatitis, blisters, abscesses or cellulitis, swelling, pressure ulcers, wound dehiscence, hematoma, and seroma.

# Expected Benefits and Risks/Discomfort Associated with Study Participation

## Expected Benefits

1. Reduction of perineal wound SSI, potentially leading to shorter hospital stay, reduced medical costs, and improved patient quality of life (QOL).
2. Prevention of delays in initiating postoperative adjuvant chemotherapy, potentially leading to improved curability of rectal cancer and enhanced long-term prognosis.

## Burdens and Potential Risks

1. Delayed detection of wound infections due to pNPWT application, potentially leading to progression of superficial incisional SSI to deep incisional SSI or organ/space SSI.
2. Skin damage caused by the application and removal of the pNPWT foam filler, including epidermal detachment and erosion.
3. Increased discomfort in the perineal and gluteal regions due to pNPWT application.

## Measures to Minimize Burdens and Risks

Regular monitoring will be conducted to evaluate adverse events and other safety concerns.

# Statistical Analysis

## Analysis Population

### Safety Analysis Set (SAS)

The safety analysis set (SAS) will include all enrolled patients who had the investigational device applied, even partially.

### Efficacy Analysis Set (EAS)

The efficacy analysis set (EAS) will include all enrolled patients who had the investigational device applied and did not violate major study protocol criteria (e.g., failure to meet inclusion criteria, lack of consent, unregistered cases, or other significant procedural violations).

## Analysis Methods

### Primary Analysis

1. Incidence of Perineal Wound SSI

The proportion of patients who develop perineal wound SSI will be calculated along with the 95% confidence interval.

### Secondary Analyses

#### Efficacy Analyses

1. Incidence of postoperative complications other than perineal wound SSI

The proportion of patients experiencing Clavien-Dindo Grade III or higher complications will be calculated along with the 95% confidence interval.

1. Postoperative length of hospital stay

The median and interquartile range of postoperative hospital stay will be calculated.

1. Completion rate of pNPWT

The proportion of patients who successfully maintained pNPWT for 7 days postoperatively will be calculated along with the 95% confidence interval.

#### Safety Analysis

The number and proportion of patients experiencing adverse events or study-related conditions will be reported.

## Target Sample Size and Rationale for Determination

The target sample size for this study is 50 patients.

< Rationale for Sample Size Calculation >

Existing reports indicate that the incidence of perineal wound SSI in patients receiving pNPWT ranges from 9.1% to 14.8%, whereas the incidence in patients not receiving pNPWT ranges from 32.6% to 41.7% ^(18,20)^. This study assumes that the incidence of perineal wound SSI within 30 days postoperatively will be 12%, with a threshold of 40% based on prior reports. Given a two-sided significance level of 5% and a power of 80%, the required sample size is 45 patients. To account for potential dropouts, the planned enrollment target is set at 50 patients.

< Estimated Patient Accrual >

The participating institutions collectively treat approximately 80 patients per year who meet the eligibility criteria. Assuming an eligibility rate of 70% and a consent rate of 70%, it is expected that 50 patients can be enrolled within 15 months.

## Changes to the Statistical Analysis Plan

If any changes are made to the statistical analysis plan, the study protocol will be revised accordingly. The revised protocol must be approved by the CRB and the institutional administrators before implementation. Any modifications will be documented in the final study report.

# Data Management

## Case Report Form (CRF) Completion and Submission

This study will use an electronic data capture (EDC) system (REDCap system) for case report form (CRF) creation and submission. Participating institutions must retain the original data used for CRF entries as source documents. Any paper-based data provided by study subjects must be verified and recorded by the participating institution.

The principal investigator or sub-investigators must enter required study data into the REDCap system and adhere to CRF entry guidelines. Any modifications or corrections must follow the established CRF entry rules. The principal investigator is responsible for ensuring that all entered data is complete and accurate.

All CRF data will be electronically stored in the REDCap database throughout the study duration. Upon completion of final data entry and corrections, copies of the CRF data, including audit logs, must be retained at each participating institution in accordance with study regulations.

## Handling of Data

If issues arise regarding enrolled cases, the principal investigator and statistical analysis lead will discuss and determine appropriate handling. The decision, along with relevant details and dates, will be recorded.

## Identification of Source Documents

The source documents supporting CRF data in this study include the following:

・Records of informed consent and study information provided to subjects

・Medical records, imaging data, and other clinical documentation supporting CRF entries

・Other study-related records maintained at participating institutions

For patient background information, adverse events, and concomitant treatments, the CRF itself will serve as the source document.

# Direct Access to Source Documents

The principal investigator and participating institutions will allow direct access to all study-related source documents for monitoring, auditing, and review by the CRB and regulatory authorities as required.

# Monitoring and Auditing

## Monitoring

To ensure the reliability of the study and the protection of study subjects, the principal investigator will appoint designated monitors to assess study progress and verify compliance with the Japanese Clinical Trials Act, relevant regulations, and the study protocol.

## Designation of Monitors

Monitors must meet the following criteria:

・Possess adequate scientific and clinical knowledge relevant to monitoring activities.

・Have a thorough understanding of the Japanese Clinical Trials Act and related regulations.

・Be well-versed in the study protocol, informed consent documents, and standard operating procedures.

・Have no direct involvement in the study’s execution.

・Maintain confidentiality of study subjects' personal information and study-related data.

## Monitoring Procedures

Monitoring will be conducted according to a separate monitoring procedures manual.

## Reporting of Monitoring Results

Monitors will document each monitoring visit in a monitoring report and submit it to the principal investigator. If any areas requiring improvement are identified, the principal investigator will be asked to take corrective action.

If necessary, the principal investigator will notify the lead investigator, who will then provide relevant information to other study investigators.

## Auditing

Since this study qualifies as a voluntary compliance study under the Japanese Clinical Trials Act, auditing will not be conducted.

# Approval and Amendments of the Study Protocol

## Approval, Modification, and Revision of the Study Protocol

Before initiating the study, the lead investigator must submit the study protocol and implementation plan for review by the CRB and obtain approval. Additionally, authorization from the institutional administrators of the participating medical institutions must be obtained. The implementation plan will also be submitted to the Ministry of Health, Labour and Welfare (MHLW).

If the study protocol is revised, the principal investigator must promptly inform all relevant personnel of the changes. Furthermore, the revised protocol must be submitted for review and approval by the CRB and the institutional administrators following the prescribed procedures. If necessary, modifications must also be reported to the Minister of Health, Labour and Welfare.

In the case of significant amendments, patient enrollment may be temporarily suspended to ensure subject protection. The revised protocol will not be used until it has been approved by the CRB and the institutional administrators and is published in the Japan Registry of Clinical Trials (jRCT), the official database managed by the MHLW.

## Deviations from the Study Protocol

The lead investigator and principal investigators at each institution must not implement any modifications to the study protocol without prior agreement from all investigators and approval from the CRB and institutional administrators.

However, if an urgent change is necessary to avoid an immediate hazard to study subjects, the principal investigator at each institution may implement deviations from the protocol after prior consultation with the lead investigator. Such deviations must be reported to the CRB and the institutional administrators as soon as possible.

# Study Completion, Early Termination, Suspension, and Resumption

## Study Completion

This study will be considered complete when all enrolled cases have completed the 30-day postoperative follow-up period, all data collection and analysis have been finalized, and the final study report has been compiled.

## Early Termination, Suspension, and Resumption of the Study

The principal investigator shall evaluate the feasibility of continuing the study under the following circumstances:

1. When critical information regarding safety or efficacy is obtained.
2. When difficulties in recruiting study participants make it unlikely to achieve the planned sample size.
3. When the study objectives are achieved before reaching the planned sample size or study duration.
4. When the number of cases of perineal surgical site infection (SSI) exceeds 20 (i.e., when the incidence of perineal SSI exceeds 40%, surpassing the previously reported incidence in the non-pNPWT group).
5. When the administrator of the implementing medical institution, upon receiving the opinion of the CRB, issues an instruction to modify the research plan, and compliance with such modification is deemed unfeasible.

If the administrator of the implementing medical institution, based on the opinion of the CRB, recommends or instructs the termination of the study, the study shall be discontinued.

If the study is terminated or suspended early, regardless of the reason, the principal investigator or sub-investigators shall promptly notify all study participants and take appropriate measures to ensure their safety, including conducting necessary medical examinations.

The principal investigator shall notify the Certified Clinical Research Review Board of the termination within 10 days of the termination date and report it to the Minister of Health, Labour and Welfare. Additionally, a report shall be submitted to the administrator of the implementing medical institution.

To resume a suspended clinical study, a report must first be submitted to the CRB, followed by obtaining approval from the administrator of the implementing medical institution. Thereafter, the study shall be reported to the MHLW before resumption.

# Reporting to Institutional Administrators of Participating Medical Institutions

The principal investigator shall report information regarding the clinical research to the administrator of the implementing medical institution in writing as follows:

・If the CRB provides any opinions, the principal investigator shall promptly report the content of such opinions.

・If the principal investigator becomes aware of the occurrence of diseases or other conditions as stipulated by the Clinical Research Act and related regulations, he/she shall report them within the period specified by the Clinical Research Act and related regulations.

・If the clinical research is found to be non-compliant with the Clinical Research Act, related regulations, or the research protocol, the principal investigator shall promptly report such non-compliance.

・If a summary of the primary endpoint report or the final study report is made public, the principal investigator shall promptly report it.

・The principal investigator shall report on the implementation status of the clinical research at least once a year.

# Study Funding and Financial Considerations

## Funding Sources and Conflicts of Interest

This study is funded by a research grant provided by the Japan Society of Laparoscopic Colorectal Surgery. At the time of study initiation, there are no conflicts of interest between the researchers and 3M, the manufacturer of the Prevena Incision Management System (IMS), which is used as the trial device in this study, that would affect the conduct or outcomes of the research. Conflict of interest matters are appropriately reviewed by the Kyoto University Clinical Research Conflict of Interest Review Committee in accordance with the Kyoto University Conflict of Interest Policy and the Kyoto University Conflict of Interest Management Regulations.

## Research-Related Costs, Participant Burden, and Compensation

The costs associated with the treatment in this trial will be covered within the scope of health insurance, as in standard clinical practice. No compensation will be provided to participants for their participation in this study.

# Handling of Samples and Data After Study Completion

The principal investigator and individuals involved in this study shall properly store the records related to the implementation of this study for a period of 10 years from either the date when the end of the study is reported or the date when the final publication of the study results is reported, whichever is later. After the retention period has passed, materials and information will be disposed of by shredding paper records and permanently erasing electronic data.

In order to maintain the confidentiality of the research participants, medical records managed by the hospital, other clinical data, records of consent from research participants, the correspondence table between research participants' personal information and their identification codes, as well as case report forms and equivalent documents, will be stored at the implementing medical institution.

For secondary use of the data collected in this clinical study, the research participants' consent will be obtained through a written explanatory document. Additionally, separate approval from the certified clinical research review committee, etc., will be obtained. If new specific purposes for use arise, information regarding these purposes will be communicated to or made available to the research participants, and they will be provided the opportunity to withdraw their consent for the study.

# Attribution of Research Outcomes and Publication of Results

## Attribution of Research Outcomes

The outcomes obtained from this research belong to Kyoto University.

## Method of Public Disclosure of Research Information

The overview of this research will be published on jRCT before the study begins. The published content will be updated as needed in response to amendments to the study protocol or the progress of the research. Additionally, an outline of the primary outcome report or the final study report summarizing the results will be made publicly available.

If the research findings are to be presented in a paper or at an academic conference, the decision will be made through consultation among the principal investigators at each participating medical institution and the responsible statistician, among others.

# Study Implementation Period

Participant Enrollment Period: From the date of study protocol publication to February 28, 2026 Participant Observation Period: From the date of study protocol publication to March 31, 2026

Study Implementation Period: From the date of study protocol publication to May 31, 2026

# Study Organization

1. Principal Investigator

The principal investigator holds comprehensive responsibility for the planning, execution, and overall management of this study.

Kazutaka Obama

Professor, Department of Surgery, Kyoto University Hospital

Address: 54 Kawahara-cho, Shogoin, Sakyo-ku, Kyoto 606-8507, Japan

Phone: +81-75-366-7595

Fax: +81-75-366-7642

1. Data Management Supervisor

Yoshiro Itatani

Associate Professor, Department of Surgery, Kyoto University Hospital

Address: 54 Kawahara-cho, Shogoin, Sakyo-ku, Kyoto 606-8507, Japan

Phone: +81-75-366-7595

Fax: +81-75-366-7642

1. Monitoring Responsible Person

Ryosuke Okamura

Assistant Professor, Department of Surgery, Kyoto University Hospital

Address: 54 Kawahara-cho, Shogoin, Sakyo-ku, Kyoto 606-8507, Japan

Phone: +81-75-366-7595

Fax: +81-75-366-7642

1. Monitor

Ryo Ohno

Graduate Student, Department of Surgery, Graduate School of Medicine, Kyoto University

Address: 54 Kawahara-cho, Shogoin, Sakyo-ku, Kyoto 606-8507, Japan

Phone: +81-75-366-7595

Fax: +81-75-366-7642

Takuya Takami

Graduate Student, Department of Surgery, Graduate School of Medicine, Kyoto University

Address: 54 Kawahara-cho, Shogoin, Sakyo-ku, Kyoto 606-8507, Japan

Phone: +81-75-366-7595

Fax: +81-75-366-7642

1. Head of Statistical Analysis

Nobuaki Hoshino

Associate Professor, Institute for Advancement of Clinical and Translational Science,

Kyoto University Hospital

Address: 54 Kawahara-cho, Shogoin, Sakyo-ku, Kyoto 606-8507, Japan

Phone: +81-75-751-4748

Fax: +81-75-751-4746

1. Research Secretariat

Yoshiro Itatani

Associate Professor, Department of Surgery, Kyoto University Hospital

Address: 54 Kawahara-cho, Shogoin, Sakyo-ku, Kyoto 606-8507, Japan

Phone: +81-75-366-7595

Fax: +81-75-366-7642

Takuya Takami

Graduate Student, Department of Surgery, Graduate School of Medicine, Kyoto University

Address: 54 Kawahara-cho, Shogoin, Sakyo-ku, Kyoto 606-8507, Japan

Phone: +81-75-366-7595

Fax: +81-75-366-7642

Participating institutions in the Study and the principal investigators at each institution

The principal investigator at each institution is responsible for the planning, execution, and operational management of this study at their respective institution.

1. Fukuoka University Hospital

Suguru Hasegawa

Professor, Department of Gastroenterological Surgery, Fukuoka University Hospital

Address: 7-45-1 Nanakuma, Jonan-ku, Fukuoka 814-0180, Japan

Phone: +81-92-801-1011

1. Kindai University Hospital

Junnichiro Kawamura

Professor, Department of Surgery, Kindai University Hospital

Address: 377-2 Onohigashi, Osakasayama-shi, Osaka 589-8511, Japan

Phone: +81-72-366-0221

1. Tokai University Hospital

Seiichiro Yamamoto

Professor, Department of Gastroenterological Surgery, Tokai University Hospital

Address: 143 Shimokasuya, Isehara-shi, Kanagawa 259-1193, Japan

Phone: +81-463-93-1121

1. Kanazawa University Hospital

Noriyuki Inaki

Professor, Department of Surgery, Kanazawa University Hospital

Address: 13-1 Takara-machi, Kanazawa, Ishikawa 920-8641, Japan

Phone: +81-76-265-2000

1. University Hospital, Kyoto Prefectural University of Medicine

Hiroki Shimizu

Assistant Professor, Department of Digestive Surgery,

University Hospital, Kyoto Prefectural University of Medicine

Address: 465 Kawaramachi-Hirokoji, Kamigyo-ku Kyoto 602-8566, Japan

Phone: +81-75-251-5111

1. Osaka Red Cross Hospital

Akinari Nomura

Co-director, Department of Gastrointestinal Surgery, Osaka Red Cross Hospital

Address: 5-30 Fudegasakicho, Tennouji-ku, Osaka 543-8555, Japan

Phone: +81-6-6774-5111

1. Medical Research Institute KITANO HOSPITAL, PIIF Tazuke-Kofukai

Takehito Yamamoto

Staff Physician, Department of Gastrenterology Surgery,

Medical Research Institute KITANO HOSPITAL, PIIF Tazuke-Kofukai

Address: 2-4-20 Ohgimachi, Kita-ku, Osaka 530-8480, Japan

Phone: +81-6-6361-0588

1. Kobe City Nishi-Kobe Medical Center

Atsushi Itami

Director, Department of Surgery, Kobe City Nishi-Kobe Medical Center

Address: 5-7-1 Kojidai, Nishi-ku, kobe-shi, Hyogo 651-2273, Japan

Phone: +81-78-997-2200

1. Shiga General Hospital

Masahiro Yamada

Director, Department of Surgery, Shiga General Hospital

Address: 5-4-30 Moriyama, Moriyama-shi, Shiga 524-8524, Japan

Phone: +81-570-00-5031

# Certified Clinical Research Review Board

Kyoto University Certified Review Board

Certification Number: CRB5180002

Address: Yoshida-Konoe-cho, Sakyo-ku, Kyoto 606-8501, Japan

Phone: +81-75-753-4680

Email: ethcom@kuhp.kyoto-u.ac.jp

# References

(1) Fan,C.W. et.al: Int J Colorectal Dis. 2023.38(1):p.73. [PMID: 36933148]

(2) Althumairi,A.A. et.al: World J Surg. 2016.40(7):p.1755-1762. [PMID:26908238]

(3) Salmenkyla,T. et.al: BMC Surg. 2022.22(1):p.296.

(4) Musters,G.D. et.al: Int J Colorectal Dis. 2014.29(9):p.1151-1157. [PMID:25064389]

(5) Wiatrek,R.L. et.al: Clin Colon Rectal Surg. 2008.21:p.76–85.

(6) Stone,H.B. et.al: Lancet Oncol. 2003.4:p.529-536.

(7) Hultman,C.S. et al: Ann Plast Surg. 2010.64(5):p.559-62. [PMID:20395804]

(8) Benedict,K.C. et.al: J Plast Reconstr Anesthet Surg. 2023.84:p.514-520. [PMID:37418850]

(9) Gultekin,S. et.al: ANZ J Surg. 2022.92(11):p.2968-2973.

(10) Howell,A.M. et.al: Int J Surg. 2013.11:p.514-517.

(11) Butt,H.Z. et al: Int J Colorectal Dis. 2013.28:p.1459-1468.

(12) Morykwas,M.J. et.al: Ann Plast Surg. 1997.38(6):p.533-562. [PMID:9188970]

(13) Walma,M.S. et.al: Int J Surg. 2016.26:p.18-24.

(14) Komono,A et.al: Surg Case Rep. 2022.8(1):p.116. [PMID:35718851]

(15) 2018 Perioperative Management Guidelines for SSI Prevention in Gastrointestinal Surgery, SHINDAN TO CHIRYO SHA Inc, Tokyo, 2018.

(16) Wilkes,R.P. et.al: Surg Innov. 2012.19:p.67-75.

(17) Meeker,J. et.al: J Orthop Trauma. 2011.25:p.755-761.

(18) Kaneko,T. et.al: Int World J. 2021.18(1):p.103-111.

(19) Javed,A.A. et.al: Ann Surg. 2019.269(6):p.1034-1040.

(20) Meyer,J. et.al: Int J Colorectal Dis. 2021.36(1):p.19-26. [PMID:32886194]

(21) Horan,T.C. et.al: Am J Infect Control. 2008.36(5):p.309-332. [PMID:18538699]

(22) Japanese Classification of Colorectal, Appendiceal, and Anal Carcinoma, 9th Edition, KANEHARA & CO., LTD, Tokyo, 2022.

# Appendix

Appendix 1: Information Document

Appendix 2: Informed Consent Form

Appendix 3: Procedures for Handling the Occurrence of Diseases and Other Adverse Events

Appendix 4: Conflict of Interest Management Plan

Appendix 5: Accompanying Documentation for the Investigational Device

Appendix 6: Monitoring Procedures
